# Experience implementing a university-based mass immunization program in response to a meningococcal B outbreak

**DOI:** 10.1080/21645515.2018.1547606

**Published:** 2019-01-08

**Authors:** Blair Capitano, Krista Dillon, Andre LeDuc, Bruce Atkinson, Cynthia Burman

**Affiliations:** a Vaccines US Medical Affairs, Pfizer Inc, Collegeville, PA, USA; b Emergency Management & Continuity, University of Oregon, Eugene, OR, USA; c Safety and Risk Services, University of Oregon, Eugene, OR, USA; d Medical Development, Scientific & Clinical Affairs, Pfizer Vaccines, Collegeville, PA, USA

**Keywords:** meningococcal meningitis, serogroup B, outbreak, college campus, student, mass immunization clinic, United States

## Abstract

*Neisseria meningitidis* serogroup B (MenB) has caused several recent outbreaks of meningococcal disease on US college campuses. In January 2015, a case of MenB was reported at a university in Oregon, culminating in an outbreak with a total of 7 cases (including 1 fatality) identified over a 5-month period. In response to the outbreak, the university organized a mass immunization campaign with 4 “opt-in” immunization clinics. The preparation, challenges, and resources required for organization and implementation of a mass immunization program in response to an outbreak at a large public university are discussed herein. Based on the logistical challenges as well as resource expenditures associated with planning and executing a mass immunization effort, this experience illustrates that proactive, routine immunization of incoming students is the best strategy for MenB outbreak prevention.

## Introduction

*Neisseria meningitidis* serogroup B (MenB) is a pathogen that causes invasive meningococcal disease (IMD), a devastating disease associated with isolated cases as well as outbreaks in the United States.^1^ Overall, the incidence of IMD due to MenB is low in the United States, with the highest incidence rate occurring in adolescents and young adults aged 16 to 23 years in 2016 (0.12 cases per 100,000 population).^2^ When compared with other disease-causing serogroups in this age group, MenB accounts for the majority of infections (57%).^2^ MenB outbreaks are unpredictable, may be prolonged, and are associated with substantial morbidity and mortality.^3,4^

MenB has been responsible for several recent US college campus–based outbreaks.^5,6^ For example, between March 2013 and March 2014, 9 cases were confirmed at a university in New Jersey, with 1 of the cases occurring at a nearby university after social mixing^7^; 5 cases were confirmed at a university in California beginning in November of 2013.^1,8-10^ The duration of these outbreaks was variable, with some cases clustered over a short time frame, and others identified over a prolonged period.^11^ Importantly, at the time of these outbreaks in 2013–2014, no vaccine for protection against MenB disease was available in the United States. In response to the unmet need for MenB immunization, the US Food and Drug Administration (FDA) granted investigational new drug status for mass immunization on the university campuses for a MenB vaccine formerly approved elsewhere, but not FDA-approved for use in the United States (MenB-4C, Bexsero®, 4CMenB; GlaxoSmithKline Vaccines, Srl, Siena, Italy).^1,12^ Subsequently, the FDA granted accelerated approval for MenB-FHbp (Trumenba®, bivalent rLP2086; Pfizer Inc, Philadelphia, PA)^13^ in October 2014 and MenB-4C in January 2015, for active immunization to prevent MenB disease in individuals 10 through 25 years of age.^13-15^

The Advisory Committee on Immunization Practices (ACIP) recommendations for the use of MenB vaccines were first published in June 2015.^8^ The ACIP recommendations are “tiered” and include a category A component for individuals aged ≥ 10 years at increased risk of IMD and a category B component for all adolescents and young adults 16 through 23 years of age.^1^ In October 2015, the ACIP published recommendations that a MenB vaccine series may be administered to adolescents and young adults aged 16 through 23 years to provide short-term protection against most strains causing MenB disease (category B), with the preferred age for immunization being between 16 and 18 years.^8^ This additional recommendation is designed to protect adolescents and young adults during the period of increased risk (ie, when disease incidence in the United States in adolescents/young adults peaks, between 18 and 23 years of age).^1^

In early 2015, 2 additional outbreaks of MenB disease occurred on US college campuses: 7 cases at a university in Oregon (described herein as “the University”) starting in January 2015, and 2 cases at a college in Rhode Island in February 2015.^5,16^ In response to these outbreaks, campus-based immunization campaigns were undertaken in an effort to reduce MenB transmission at both the University (reported herein) and the college in Rhode Island.^17^

The current article focuses on the MenB outbreak experience at the University. The objectives of this review are to (1) describe the response to a MenB disease outbreak on a college campus, including the real-world use of MenB-FHbp as part of a mass immunization program; (2) identify the actions, challenges, and resources required to implement a campus-based mass immunization program at a large public university; and (3) discuss proactive, routine immunization as a key strategy for prevention of future MenB disease outbreaks.

## Results

### Overview of the university

This university is located in Oregon on an urban campus of 295 acres. The student population is approximately 24,125 (including 20,552 undergraduate and 3573 graduate students) of which 16% live in residence halls. An on-campus Student Health Center provides basic health services to enrolled students. The University Emergency Management program includes an Incident Management Team (IMT) that is activated for emergencies, including communicable disease outbreaks.

### Meningococcal serogroup B cases

The MenB outbreak at the University comprised 7 cases, beginning in mid-January 2015 and lasting through May 2015 (**Supplemental Figure**). A fourth case (a fatality) occurred 33 days after the initial case was reported. Four days later, a MenB outbreak was declared by state Health officials and subsequently confirmed by the Centers for Disease Control and Prevention (CDC). After initiation of the campus-based mass immunization program on day 47, 3 additional cases were confirmed. Two cases occurred between the first and second immunization clinic. Based on 6 cases identified in students, the attack rate was 30.5 per 100,000 undergraduate students. After the second immunization clinic, 1 case of MenB was reported in a father who had visited the campus approximately 4 months after the first case was reported.

### Laboratory investigation

Local testing was performed at the treating hospital, which typically included Gram staining of cerebrospinal fluid and/or blood and PCR in some cases. Latex agglutination for initial serogrouping was performed at associated laboratories and confirmatory serogroup testing at the Oregon State Public Health Laboratory. Isolates were forwarded to the CDC for further molecular characterization, which indicated that all the strains from the 7 cases were ST-32/CC32.^18^

### Mass immunization/emergency response

A timeline of the extensive coordination and communication efforts, and actions implemented by the University during the first 3 months of the outbreak, is outlined (**Supplemental Figure**). Specifically, within 48 hours of notification of the first confirmed MenB case, the University activated the IMT to assist in the coordination of responses initiated by staff from the Emergency Management program. Team members included an Incident Commander, Public Information Officers, and representatives from the University Health Center and the Registrar’s Office. The IMT implemented a standard protocol developed to address the identification and confirmation of a meningitis case in a university student. Multiple activities defined in this protocol included coordination with the County Public Health Department; notification of close contacts, including roommates, classmates, coworkers, and faculty members; information updates on the Student Health Center website, such as information about signs and symptoms of meningitis; administration of prophylactic antibiotics to close contacts; notifications to university officials; and implementation of additional campus-wide communication plans.

This standard protocol was implemented following each of the 7 cases of MenB that occurred at the University. After the second MenB case was confirmed, University and County Public Health Department staff engaged in planning discussions for large-scale immunization clinics, and several meetings were held to evaluate potential locations, vaccine sources, and staffing. After the third MenB case was confirmed, the CDC threshold in place at that time was met for declaration of an outbreak (≥ 3 confirmed or probable cases within the same serogroup within ≤ 3 months with an attack rate of 10 cases per 100,000, which differs to the current threshold for an institutional outbreak of 2–3 outbreak-associated cases within ≤ 3 months).^19,20^ However, an outbreak was not declared at this stage because cases 2 and 3 were determined to be close contacts. Following this third case, a campus-wide email message was sent to all students, faculty and staff articulating the signs and symptoms of MenB disease and preventive actions. Suggested proactive strategies to prevent infection included immunization, as well as washing hands, reducing the spread of aerosolized bacteria, and refraining from sharing personal items. Similar information was sent to the Parents’ Association, an “opt-in” group for parents of students at the University. It is important to note that universities generally do not collect and store parent contact information, as the majority of students entering as freshman are ≥18 years of age. Two days after the fourth MenB case resulted in a fatality, an outbreak was officially declared, and the University received a joint recommendation from the CDC, the state Health Authority, and County Public Health officials to implement a clinic to vaccinate approximately 22,000 students at the earliest opportunity. Immunization was recommended by the state Health Authority for current and incoming undergraduate students, graduate students, faculty, and staff who lived or planned to live in residence halls, fraternities, and sororities.^21^

Because the 2 MenB vaccines, MenB-FHbp and MenB-4C, were only recently approved by the FDA and new to the market, they were not readily available through county or state vaccine provision mechanisms; therefore, funding was not available to offset vaccine purchase costs that the University would incur. At the suggestion of the State, the University contacted both manufacturers, requesting pricing information and the option for a fully supported clinic that could provide support for immunization-related services, because neither the University nor the county or state possessed the resources to implement a large-scale clinic. Because of the time-sensitive nature of the request, the University stipulated that proposals should be received within 48 hours. Ultimately, MenB-FHbp was selected based on the proposal to include a partnership with pharmacies located within the community; these locations served to complement the mass immunization clinics held at the University. MenB-FHbp was available at several local pharmacies, and could be invoiced to insurance, thus ensuring that students would not receive a payment request prior to vaccine administration; such requests could ultimately limit the rate of pharmacy-based immunization during the outbreak. The ultimate goal was to eliminate as many barriers to immunization as possible. In addition to vaccine acquisition, the pharmacies were familiar with vaccine storage procedures and logistics, which included providing additional refrigerators as well as a refrigerated truck that was present on site during the immunization clinics.

In total, 4 mass immunization clinics were held on campus on days 47–50, 118–120, 264–265 and approximately 13 months after the initial case was diagnosed. Additional, smaller immunization clinics also took place. Details pertaining to the immunization efforts, including daily doses administered to the student body, are reported in Table 1. All enrolled students were informed of the availability of vaccine through email messages sent through the University’s emergency notification system. In addition, a marketing and outreach immunization campaign was developed that included materials, such as images of student leaders and athletes with shirt sleeves rolled up to reveal bandages with university logos. These images were included in posters and table tents strategically placed throughout public campus spaces, including housing, dining halls, and the Student Union building. Faculty received a PowerPoint (Microsoft, Redmond, WA) presentation for display at the beginning of classes to inform students of the various clinic locations and hours of operation. The same slides were used for digital displays in several buildings across campus. Notifications of immunization clinics were posted in the University’s class registration system, ensuring students viewed clinic details when logging into the system. Advertisements were also placed in the student newspaper.

**Table 1.** Details of MenB immunizations during the 2015 outbreak at the university.

MenB^a^ Immunizations				
Day After Case 1 Diagnosis	Location	Event	Daily Doses, n	Cumulative Doses, n
40	Sports arena, UHC	Surge Clinic	503	503
41	Sports arena, UHC	Surge Clinic	678	1181
42	Sports arena, UHC	Surge Clinic	666	1847
43	Sports arena, UHC	Surge Clinic	638	2485
44	Sports arena, UHC	Surge Clinic	788	3273
47	Sports arena, pharmacy, UHC	Mass Immunization Clinic	898	4171
48	Sports arena, pharmacy,^b^ UHC	Mass Immunization Clinic	1110	5281
49	Sports arena, pharmacy, UHC	Mass Immunization Clinic	203	5484
50	Sports arena, pharmacy,^b^ UHC	Mass Immunization Clinic	249	5733
57	Off-campus	Clinic	78	5811
58	Off-campus	Clinic^c^	181	5922
83	On-campus	Clinic	126	6118
84	On-campus	Clinic	186	6304
85	On-campus	Clinic	337	6641
90	On-campus	Mini-clinic	17	6658
91	On-campus	Mini-clinic	0	6658
118	Sports arena, pharmacy, UHC	Mass Immunization Clinic	637	7295
119	Sports arena, pharmacy,^b^ UHC	Mass Immunization Clinic	774	8069
120	Sports arena, pharmacy, UHC	Mass Immunization Clinic	1304	9373
163	On-campus	Orientation clinic	163	9536
167	On-campus	Orientation clinic	199	9735
169	On-campus	Orientation clinic	17	9752
180	On-campus	Orientation clinic	169	9921
184	On-campus	Orientation clinic	202	10,123
187	On-campus	Orientation clinic	184	10,307
191	On-campus	Orientation clinic	180	10,487
195	On-campus	Orientation clinic	20	10,507
198	On-campus	Orientation clinic	158	10,665
202	On-campus	Orientation clinic	151	10,816
264–265	Sports arena, pharmacy,^b^ UHC	Mass Immunization Clinic	2641	13,457
397–398	Sports arena, pharmacy, UHC	Mass Immunization Clinic	1198	14,665

UHC = University Health Center.

^a^MenB-FHbp and MenB-4C combined total.

^b^Participating pharmacies included Safeway, Walgreens, and Albertsons.

^c^Apartment complex of Case 5.

In addition to the mass immunization clinics, several smaller clinics were held during summer freshman orientation to allow incoming freshman to receive 1 dose before their arrival on campus (Table 1). In the fall of 2015, immunization information was readily available during freshman orientation and included information for parents.

### Mass immunization clinic logistics

The mass immunization clinics took place at the campus sports arena. This facility provided sufficient space to allow for the several sequential checkpoints and stations required for appropriate handling and flow of approximately 22,000 eligible students. The checkpoints were staffed by key IMT members from Emergency Management, Athletics, Communications, and the Student Health Center. Approximately 30 staff volunteers from the University across a variety of departments participated in each clinic shift; approximately 2000 person-hours were logged by staff over the course of the 4 clinics.

Specific details of the stations at each clinic and activities performed are outlined in Table 2. Briefly, before students were vaccinated, their immunization history was reviewed and eligibility for MenB vaccine receipt was confirmed. Forms pertaining to insurance were completed, along with informed consent forms for a CDC nasopharyngeal carriage study for students willing to participate. Medical information pertaining to MenB immunization was provided in the form of a 1-page informational flier. In addition, students had the option of speaking with a pharmacist to address any questions prior to being immunized. Medical Affairs personnel, employed by the vaccine manufacturer, were on site to provide medical information support to the local pharmacists who were administering the MenB vaccine. After immunization, students were directed to a rest area and provided an additional opportunity to participate in the nasopharyngeal carriage study. Small gift items were provided by the CDC for participation in the study. The University offered a variety of food and gift incentives to motivate student attendance at the clinics. Student apathy towards MenB disease prevention ultimately proved to be the greatest challenge to maximum participation in the immunization campaign; considerable efforts were undertaken via both health marketing and communication to address this challenge.

**Table 2.** Sequential list of stations employed during mass immunization clinics at the university.

Station	Activity
Immunization check	Pharmacist cross-checked student immunization history with the state vaccine database to determine prior MenB immunization history.
Eligibility check	University employee volunteers confirmed University enrollment for vaccinees.
Forms	Students completed consent forms and were invited to participate in a behavioral study conducted by the State Health Authority.
Form check	Accuracy of consent forms were checked for insurance billing purposes.
Insurance station	Eligible students without health insurance were enrolled in the state-sponsored health plan. Donated vaccine was made available to students without health insurance if ineligible for enrollment in the state-sponsored health plan.
Medical station	Information was provided to students with medical questions about the vaccine. Consultation was provided to students based on responses to a series of prescreening questions.
Immunization station	Pharmacists advised students about potential side effects and completed immunization.
Rest area	Students were advised to rest for 15 minutes following their immunization; volunteers with basic medical experience were available, and snacks and water were provided. Students were invited to participate in a carriage study conducted by the CDC.
CDC carriage study station registration	Nasopharyngeal swabs were collected for meningococcal carriage study. Gift cards were provided to volunteers.
Swag table	Swag items were gifted to encourage participation in upcoming clinics; items included wristbands, pizza, and gift cards redeemable at the University Book Store.

CDC = Centers for Disease Control and Prevention.

Student participation in the mass immunization clinics was “opt-in,” meaning there was no requirement for student participation from the University. During the first 2 mass immunization clinics, ≤ 1304 students were immunized per day, and 5175 immunizations were administered overall. By mid-April, approximately 6660 students had received a dose of MenB vaccine. During the outbreak, both approved MenB vaccines were available at the Student Health Center as well as local area provider locations, with MenB-FHbp received by the majority of students (Table 1). MenB-FHbp was administered as a 3-dose schedule; receipt by all eligible students was approximately 60% for dose 1, 29% for dose 2, and 10% for dose 3. Approximately half of the immunized students received their vaccines at the on-campus clinics, with the remaining vaccines administered at local community pharmacies and off-campus clinics.

### Resources required for the outbreak response

Meningococcal outbreaks are not easily controlled once they occur. They require coordination and advanced planning among multiple stakeholders, as well as significant human and capital resources. Communication with college-aged students can be difficult due to a myriad of competing priorities, further emphasizing the need for concise and direct public health–related messaging for this age group. Outreach campaigns should include input and participation from students or peer-to-peer groups to assist public health and university officials with optimal translation of public health information into modes and formats that will appeal to the general student population. Effective coordination of communication, education, awareness, and counseling, in addition to procurement and storage of vaccine, mass immunization strategies, vaccine clinic staffing, and administrative support, are among the more daunting logistical issues that require effective management and expedited execution during meningococcal outbreaks. The **Supplemental Figure** provides a detailed timeline of the evolution of the coordinated response to the reported MenB cases at the University, and the corresponding actions taken in response to this rapidly evolving public health threat.

For financial resource expenditure, the out-of-pocket cost of the MenB outbreak at the University was estimated to be $589,800. This amount represents the cost of the vaccine (90%), logistics (6%), marketing (2%), and partner coordination (< 1%). For the University, vaccine procurement represented a majority of the total cost outlay for outbreak management. The uniqueness of this challenge was posed primarily by the timeframe of the outbreak, which occurred very shortly after FDA approval of the vaccine but prior to broad-based insurance coverage. Overcoming this challenge required numerous hours of communication with multiple insurance companies, and ultimately, insurance coverage was provided for approximately 80% of those vaccinated. Lack of health insurance; student ineligibility at the time of immunization (eg, students had insurance but coverage had lapsed), a requirement for primary medical care provider-administered immunization; and absence of a pharmacy benefit under some insurance plans collectively accounted for the approximately 20% of individuals for whom vaccines were not covered. For students whose insurance plans lacked a pharmacy benefit and who were vaccinated at a local pharmacy, the vaccine manufacturer contacted payers for approval. The local pharmacy was not credentialed or contracted for vaccines under all payers’ medical benefits; therefore, full reimbursement was not attained in some cases.

In addition to vaccine procurement–related expenses, other essential and considerable resources required for the execution of a mass immunization clinic included, but were not limited to, university personnel person-hours; immunizers and medical personnel (eg, pharmacist immunizers, medical support, and administrative support); marketing (eg, health promotion and communication materials); rental costs for a facility that provided adequate space for the clinic; and vaccine product management and logistics (eg, transportation, refrigeration, administration cost, and supplies). These resources account for the remaining 10% of outbreak-related expenditures.

## Discussion

The benefits of immunization programs, such as that conducted at the university in Oregon, ultimately depend on the rate of infection spread and the ability of vaccines to interrupt carriage and transmission.^11,22^ For *N meningitidis*, transmission occurs rapidly,^11^ resulting in a narrow, early window in which to effectively blunt the outbreak by mass immunization.^22^ Therefore, it is important for colleges and universities to have proactive strategies to minimize this risk of a MenB outbreak on campus.

Currently, a proactive immunization approach is implemented to prevent meningococcal disease caused by serogroups A, C, Y and W, with many universities and colleges requiring incoming students to be immunized.^23^ This approach has been effective as evidenced by the fact that recent campus meningococcal outbreaks have been caused by MenB.^24^ Although similar forward-thinking strategies using proactive immunization against MenB was evident at a university in North Dakota, where in 2018 the university organized a free MenB immunization clinic in the absence of a case on campus for all students 18 to 23 years of age,^25^ proactive, campus-wide, MenB immunization strategies for students are atypical. As such, MenB cases continue to emerge on college and university campuses. In 2017 alone, confirmed or presumed cases of MenB have been reported on a number of campuses, including 2 campuses in Massachusetts,^26,27^ 1 campus in Oregon,^28^ and 1 campus in Pennsylvania.^29^ Additionally, outbreaks have been declared on a campus in Oregon^28^ and Massachusetts;^30^ immunization was offered in response to these campus outbreaks.^27,28^ Based on the geographic proximity to an outbreak or the occurrence of an isolated case, a university in Pennsylvania^29^ and Massachusetts^31^ elected to hold proactive immunization clinics targeting MenB.

In response to the identification of a MenB case on campus, expert input and active participation are required to develop a MenB outbreak control plan and to implement containment procedures. Currently, many colleges and universities are likely to be ill-equipped to manage such a comprehensive, rapid, and targeted public health response. Additional and unexpected circumstances were encountered in the course of the University outbreak described in this report; these included an overall lack of student concern for the immediacy of the situation evolving in the community and a general fear of potential vaccine side effects; both situations were addressed through additional measures of communication and education.

In addition to major logistical challenges for campus health and emergency planning personnel, university-based MenB outbreaks require significant expenditures, particularly when strategies are reactive. For example, the cost associated with managing the MenB outbreak at the University was approximately $600,000. To further understand the costs and public health burden associated with IMD outbreaks and the potential benefits of proactive immunization, in 2013 a systematic literature review estimated the cost for large disease containment strategies to range between $105,484 and $1,081,627, with an average cost per IMD case of $55,755.^32^ While this systematic review assessed IMD outbreaks globally and included both large- and small-scale containment strategies, substantial costs were incurred in all cases. At colleges and universities in the United States, the costs of managing an outbreak vary depending on the status of the campus as a public or private university and the availability of health insurance coverage. These estimates do not take into account lifetime costs to an IMD survivor due to clinical sequelae as well quality of life.^32^

Ultimately, from the perspective of a large public university, the best approach for the prevention of transmissible diseases, such as MenB, would be to routinely recommend immunization of incoming students prior to their arrival on campus. This approach is embodied in current ACIP recommendations for adolescent MenACWY immunization,^8^ which favors immunization for the prevention of meningococcal acquisition and carriage, and the interruption of transmission in the adolescent and young adult populations.^33^ Asymptomatic carriage rates for *N meningitidis* can be as high as 37% in adolescents and young adults aged 15 to 24 years.^34^ Because the use of recombinant MenB vaccines is relatively new, data on carriage and transmission after MenB immunization is still forthcoming. The results from the carriage study conducted during the immunization campaign at the Oregon university have been recently published; among 328 students who participated in the analysis, no statistically significant decrease in carriage was observed after MenB vaccination in a multivariate analysis.^35^A small decrease was observed during an evaluation that occurred after the summer break and was believed to be due to seasonal variation. Similar results were reported for the MenB carriage study in university students at 10 study centers in England after MenB-4C immunization.^36^ One month after immunization, MenB carriage was not significantly reduced among students, but carriage of all *N meningitidis* isolates collectively, in addition to others, was significantly reduced by 3 months postimmunization.^36^ A large study conducted in Australian students aged ≥ 14 years immunized with MenB-4C is currently underway (NCT03089086), and the results are expected to provide important information pertaining to vaccine effectiveness for those immunized as well as the unimmunized. The effect of MenB vaccines on carriage will be of particular importance to college students, since MenB immunization is currently not universally required for this group, and the student body is expected to be heterogeneous with regard to meningococcal immunization. In addition, further elucidation of the duration of protection afforded by MenB vaccines is required to better inform immunization policies, including regarding the necessity of booster dosing for at-risk populations. However, it is of note that ACIP recommends MenB vaccination be considered from the age of 16 years with the preferred age of vaccination at 16 through 18 years, and published data document persistence of protective immunity with MenB vaccines through 4 years after vaccination.^8,37,38^ When all information is assessed in total, routine (prior) immunization of university students can be reasonably viewed as a more effective proactive strategy overall compared with reactive, mass immunizations undertaken only after an outbreak has occurred, especially since MenB vaccines require ≥ 2 doses for a complete immunization series.

Administration of prophylactic antibiotics to close contacts was included as part of the standard protocol in response to the outbreak. In addition to recommendations for vaccination, the importance of prophylactic antibiotics of close contacts as a means to prevent additional cases has also been emphasized by the ACIP in their guidance for the public health management of meningococcal outbreaks.^20^ The guidance recommends that antimicrobial chemoprophylaxis of close contacts be initiated if a meningococcal outbreak is suspected and when meningococcal disease is strongly suspected based on laboratory and/or clinical criteria; confirmation of *N meningitidis* is not required prior to initiation of prophylaxis.

In addition to student apathy, the 3 critical challenges encountered by the University during the 2015 outbreak were cost, resources, and outreach. If private insurance had not reimbursed the cost of the vaccine for most of the immunized students, the University could well have been responsible for millions of dollars in vaccine procurement costs. Mobilizing the considerable resources required to manage this outbreak proved highly taxing for key IMT staff. Furthermore, it delayed and/or preempted normal, daily campus activities, since this outbreak occurred during a period when the University’s health center was originally preparing to implement several large-scale projects that required substantial IMT resources and oversight. Outreach to the student body to communicate the importance of immunization required creative strategies, driven in part by the many competing priorities faced by university students. Therefore, significant time and investment were required to develop communication plans used by the University.

Additional issues compounded those described above and likely served to negatively impact the number of MenB vaccine doses administered via campus-based clinics. For example, the large student body size (> 24,000) necessitated voluntary (ie, opt-in) rather than required (ie, opt-out) student participation. Furthermore, coordination and communication between the University, the County, and the State Health Authority could have been improved during the time frame spanning the first 4 confirmed cases, which occurred prior to outbreak declaration. This experience differs substantially from that described for another US campus outbreak of MenB disease at a college in Rhode Island.^16,17^ During this outbreak, campus health personnel utilized an opt-out campus immunization clinic approach for approximately 3700 eligible people that resulted in a much higher rate of immunization.^17^ In addition, close coordination between campus health and the State Health Authority occurred early in the process, prior to outbreak declaration.^17^ These contrasting experiences illustrate the absolute requirement for advanced planning and interagency coordination to successfully address a rapidly evolving, campus-based public health situation.

In conclusion, outbreaks of MenB disease are an ongoing and increasing concern on US college and university campuses. Recently approved vaccines for the prevention of MenB disease have been used to contain MenB outbreaks on several US campuses, including the University described herein. The necessary and extensive logistics for implementation of immunization clinics at the University required support from many groups, including federal, state, and local health experts, as well as pharmacy and vaccine manufacturer partnerships. Collectively, for the University outbreak, collaboration among these groups focused on removing barriers to immunization and encouraging student participation – both of which were extremely challenging, given the short time frame required for effective implementation. Although the mass immunization efforts were effective at preventing additional cases in students at the University, a proactive strategy of routinely recommended MenB immunization for adolescents, a subset of whom will attend college, could reduce or eliminate the need for the reactive efforts, such as those undertaken by the University, and at other college and university campuses.

### Materials and methods

Data pertaining to the cases of meningococcal infections in university students during the outbreak were obtained from a summary of incidence reports that was developed by the University. The medical accuracy of the data reported during the outbreak was overseen by the outbreak response team, which included employees of the University and individuals from the county and state public health authority. The State Health Authority operated in conjunction with the CDC. All information reported by the University in response to the outbreak conformed with local, state, and federal laws, including the federal Health Insurance Portability and Accountability Act and the Family Educational Rights and Privacy Act.

## References

[1] MacNeil JR, Rubin L, Folaranmi T, Ortega-Sanchez IR, Patel M, Martin SW. Use of serogroup B meningococcal vaccines in adolescents and young adults: recommendations of the advisory committee on immunization practices. MMWR Morb Mortal Wkly Rep. 2015;64(41):1171–1176. doi:10.15585/mmwr.mm6441a3. 26492381

[2] Centers for Disease Control and Prevention Enhanced meningococcal disease surveillance report. 2016 [accessed 2018 10 1]. https://www.cdc.gov/meningococcal/downloads/NCIRD-EMS-Report.pdf.

[3] Stephens DS, Greenwood B, Brandtzaeg P Epidemic meningitis, meningococcaemia, and *Neisseria meningitidis*. Lancet. 2007;369(9580):2196–2210. doi:10.1016/S0140-6736(07)61016-2. 17604802

[4] Dastouri F, Hosseini AM, Haworth E, Khandaker G, Rashid H, Booy R Complications of serogroup B meningococcal disease in survivors: a review. Infect Disord Drug Targets. 2015;14(3):205–212. doi:10.2174/1871526515999150320155614. 25809622

[5] Atkinson B, Gandhi A, Balmer P History of meningococcal outbreaks in the United States: implications for vaccination and disease prevention. Pharmacotherapy. 2016;36(8):880–892. doi:10.1002/phar.1790. 27332671

[6] Grogan J, Roos K Serogroup B meningococcus outbreaks, prevalence, and the case for standard vaccination. Curr Infect Dis Rep. 2017;19(9):30. doi:10.1007/s11908-017-0587-4. 28770496

[7] National Meningitis Association Serogroup B meningococcal disease outbreaks on U.S. college campuses. [accessed 2018 6 12]. http://www.nmaus.org/disease-prevention-information/serogroup-b-meningococcal-disease/outbreaks/.

[8] Folaranmi T, Rubin L, Martin SW, Patel M, MacNeil JR, Centers for Disease Control and Prevention. Use of serogroup B meningococcal vaccines in persons aged ≥10 years at increased risk for serogroup B meningococcal disease: recommendations of the advisory committee on immunization practices. MMWR Morb Mortal Wkly Rep. 2015;64(22):608–612. 26068564 PMC4584923

[9] MacNeil J, Advisory Committee on Immunization Practices. Epidemiology of serogroup B meningococcal disease. United States [accessed 2016 10 31]. http://www.nitag-resource.org/uploads/media/default/0001/02/243a06a67d8f4d6c08521e1d486325d2781c8b04.pdf.

[10] Garon D From mening to syringe: outbreak of meningococcal disease at a New Jersey university. New Jersey: Department of Health; 2013 [accessed 2018 6 12]. https://www.chicagohan.org/c/document_library/get_file?p_l_id=61826&folderId=88419&name=DLFE-499.pdf.

[11] Bilukha OO, Rosenstein N, National Center for Infectious Diseases, Centers for Disease Control and Prevention. Prevention and control of meningococcal disease. Recommendations of the Advisory Committee on Immunization Practices (ACIP). MMWR Recomm Rep. 2005;54(RR–7):1–21. 15917737

[12] Bexsero (Meningococcal Group B Vaccine) Full prescribing information. Sovicille (Italy): GSK Vaccines, Srl; 2017.

[13] Trumenba (meningococcal group B vaccine) Full prescribing information. Philadelphia (PA): Pfizer Inc; 2018.

[14] US Food and Drug Administration 1 23, 2015, approval letter - Bexsero. [accessed 2018 Jun 12]. http://www.fda.gov/BiologicsBloodVaccines/Vaccines/ApprovedProducts/ucm431446.htm.

[15] US Food and Drug Administration 10 29, 2014 Approval Letter - TRUMENBA. [accessed 2018 Jun 12]. https://www.fda.gov/biologicsbloodvaccines/vaccines/approvedproducts/ucm421034.htm.

[16] Soeters HM, McNamara LA, Whaley M, Wang X, Alexander-Scott N, Kanadanian KV, Kelleher CM, MacNeil J, Martin SW, Raines N, et al Serogroup B meningococcal disease outbreak and carriage evaluation at a college - Rhode Island. MMWR Morb Mortal Wkly Rep. 2015;64(22):606–607. 26068563 PMC4584922

[17] Fiorito TM, Bornschein S, Mihalakos A, Kelleher CM, Alexander-Scott N, Kanadanian KV, Raymond P, Sicard K, Dennehy PH Rapid response to a college outbreak of meningococcal serogroup B disease: nation’s first widespread use of bivalent rLP2086 vaccine. J Am Coll Health. 2017;65(4):294–296. doi:10.1080/07448481.2017.1285772. 28121236

[18] Granoff DM, Pollard AJ, Harrison LH 39 - Meningococcal capsular group B vaccines In: Plotkin SA, Orenstein WA, Offit PA editors. Plotkin’s Vaccines. 7th ed. Philadelphia, PA: Elsevier; 2018.

[19] Centers for Disease Control and Prevention Meningococcal disease. [accessed 2018 6 12]. http://www.cdc.gov/vaccines/pubs/pinkbook/downloads/mening.pdf.

[20] Centers for Disease Control and Prevention Guidance for the evaluation and public health management of suspected outbreaks of meningococcal disease. [accessed 2018 4 2]. https://www.cdc.gov/meningococcal/downloads/meningococcal-outbreak-guidance.pdf.

[21] Djibo S, Nicolas P, Alonso JM, Djibo A, Couret D, Riou JY, Chippaux JP Outbreaks of serogroup X meningococcal meningitis in Niger 1995-2000. Trop Med Int Health. 2003;8(12):1118–1123. doi:10.1046/j.1360-2276.2003.01126.x. 14641847

[22] Klepac P, Bjornstad ON, Metcalf CJ, Grenfell BT Optimizing reactive responses to outbreaks of immunizing infections: balancing case management and vaccination. PLoS One. 2012;7(8):e41428. doi:10.1371/journal.pone.0041428. 22899996 PMC3416818

[23] Immunization Action Coalition State information: meningococcal ACWY prevention mandates for colleges and universities. [accessed 2018 3 14]. http://www.immunize.org/laws/menin.asp.

[24] National Meningitis Association Meningococcal disease on U.S. College campuses, 2013-2017. [accessed 2018 3 14]. http://www.nmaus.org/wp-content/uploads/2017/01/College-Cases-Map.pdf.

[25] Minot State University Minot state to offer free meningococcal B vaccine. [accessed 2018 3 14]. http://www.minotstateu.edu/pio/news/2018/03/minot-state-to-offer-free-meningococcal-b-vaccine.shtml.

[26] Esten K. Smith college meningitis case confirmed as same serogroup as umass outbreak. The Massachusetts Daily Collegian. [accessed 2018 3 5]. https://dailycollegian.com/2018/03/update-smith-college-meningitis-case-confirmed-as-same-serogroup-as-umass-outbreak/.

[27] University of Massachesetts at Amherst 2018 Update on Meningitis B [accessed 2018 6 14]. http://www.umass.edu/meningitis.

[28] Terry L. OSU hit with 6th case of meningococcal disease; all students under 26 will need a vaccination. The Oregonian. [accessed 2018 2 12]. http://www.oregonlive.com/health/index.ssf/2017/12/osu_hit_with_6th_case_of_menin.html.

[29] Hess D Meet meningitis: is your student at risk? Kutztown University of Pennsylvania [accessed 2018 3 14]. https://www.kutztown.edu/Documents/health%20and%20wellness%20services/Website-MenBParentLetter.pdf.

[30] Centers for Disease Control and Prevention Meningococcal outbreaks. [accessed 2017 10 18]. https://www.cdc.gov/meningococcal/outbreaks/index.html.

[31] Smith College Meningitis: CDC update and second vaccination clinic. [accessed 2018 6 14]. https://www.smith.edu/news/meningitis-update-student-vaccination-clinic-thursday-march-1/.

[32] Anonychuk A, Woo G, Vyse A, Demarteau N, Tricco AC The cost and public health burden of invasive meningococcal disease outbreaks: a systematic review. Pharmacoeconomics. 2013;31(7):563–576. doi:10.1007/s40273-013-0057-2. 23673904 PMC3691489

[33] Jeppesen CA, Snape MD, Robinson H, Gossger N, John TM, Voysey M, Ladhani S, Okike IO, Oeser C, Kent A, et al Meningococcal carriage in adolescents in the United Kingdom to inform timing of an adolescent vaccination strategy. J Infect. 2015;71(1):43–52. doi:10.1016/j.jinf.2015.02.006. 25709085

[34] Yazdankhah SP, Caugant DA Neisseria meningitidis: an overview of the carriage state. J Med Microbiol. 2004;53(Pt 9):821–832. doi:10.1099/jmm.0.45529-0. 15314188

[35] McNamara LA, Thomas JD, MacNeil J, Chang HY, Day M, Fisher E, Martin S, Poissant T, Schmink, SE, Steward-Clark E, et al Meningococcal carriage following a vaccination campaign with MenB-4C and MenB-FHbp in response to a university serogroup B meningococcal disease outbreak-Oregon, 2015-2016. J Infect Dis. 2017; 216(9):1130–1140. doi:10.1093/infdis/jix446. 28968661 PMC5724767

[36] Read RC, Baxter D, Chadwick DR, Faust SN, Finn A, Gordon SB, Heath PT, Lewis DJ, Pollard AJ, Turner DP, et al Effect of a quadrivalent meningococcal ACWY glycoconjugate or a serogroup B meningococcal vaccine on meningococcal carriage: an observer-blind, phase 3 randomised clinical trial. Lancet. 2014; 384(9960):2123–2131. doi:10.1016/S0140-6736(14)60842-4. 25145775

[37] Patton ME, Stephens D, Moore K, MacNeil JR Updated recommendations for use of MenB-FHbp serogroup B meningococcal vaccine — Advisory Committee on Immunization Practices, 2016. MMWR Morb Mortal Wkly Rep. 2017;66(19):509–513. doi:10.15585/mmwr.mm6619a6. 28520709 PMC5657641

[38] Marshall HS, Richmond PC, Beeslaar J, Jiang Q, Jansen KU, Garces-Sanchez M, Martinon-Torres F, Szenborn L, Wysocki J, Eiden J, et al Meningococcal serogroup B-specific responses after vaccination with bivalent rLP2086: 4 year follow-up of a randomised, single-blind, placebo-controlled, phase 2 trial. Lancet Infect Dis. 2017; 17(1):58–67. doi:10.1016/s1473-3099(16)30314-0. 27745812

