# Supplementary material for: Experience implementing a university-based mass immunization program in response to a meningococcal B outbreak
Source: Hum Vaccin Immunother. 2019 Jan 8;15(3):717–24. doi: 10.1080/21645515.2018.1547606 (PMC6988882; doi:10.1080/21645515.2018.1547606)
Supplement: Supplemental Material [file khvi-15-03-1547606-s001.zip › Capitano_Supplemental Figure.docx]

**Supplemental Materials**


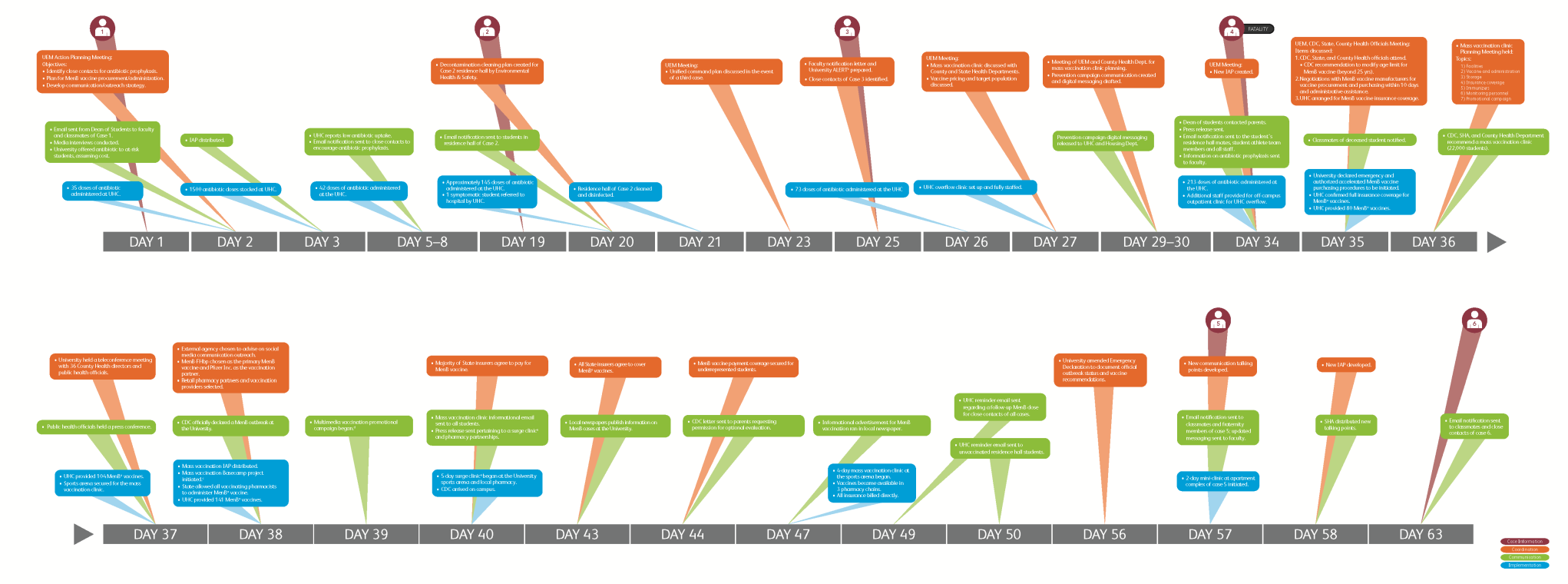


**Supplemental Figure. Timeline of University Responses After Meningococcal Group B Case Identification**

CDC=Centers for Disease Control and Prevention; IAP=Incident Action Plan; MenB=meningococcal group B; SHA=State Health Authority; UEM=University Emergency Management; UHC=University Health Center.

^a^Vaccines were MenB-FHbp and 4CMenB.

^b^ALERT is the state vaccine database. Before immunization, students were checked for prior meningococcal group B immunization.

^c^Basecamp is a project management tool that was used to share information and coordinate the response throughout the outbreak.

^d^Social media campaign aimed toward increasing immunization during the outbreak.

^e^Surge clinics occurred rapidly and without significant student marketing in order to expedite immunization.
